# Fatal Necrotizing Enterocolitis in Neonate Caused by *Cronobacter sakazakii* Sequence Type 64 Strain of CRISPR Sublineage b

**DOI:** 10.3201/eid2909.230537

**Published:** 2023-09

**Authors:** Haiyan Zeng, Chengsi Li, Jumei Zhang, Bingshao Liang, Hanjie Mei, Qingping Wu

**Affiliations:** Guangdong University of Technology, Guangzhou, China (H. Zeng);; Institute of Microbiology, Guangzhou (H. Zeng, C. Li, J. Zhang, Q. Wu);; Guangzhou Women and Children’s Medical Center, Guangzhou (B. Liang)

**Keywords:** Cronobacter sakazakii, bacteria, neonate, fatal necrotizing enterocolitis, sequence type 64, ST64, sublineage b, clustered regularly interspaced short palindromic repeats, CRISPR, single-nucleotide polymorphisms, whole-genome sequencing, antimicrobial resistance, food safety, enteric infections, China

## Abstract

We report fatal neonatal necrotizing enterocolitis in China caused by *Cronobacter sakazakii* capsular profile K1:CA1, sequence type 64, and CRISPR type 197. Phylodynamic analyses indicated that the strain originated from the ancient, widespread, and antimicrobial drug–sensitive CRISPR sublineage b. Enhanced surveillance and pathogenesis research on this organism are required.

*Cronobacter sakazakii* is a major foodborne pathogen that is associated with outbreaks of life-threatening necrotizing enterocolitis, meningitis, and sepsis in neonates and infants. Although the incidence of this pathogen is low, the case-fatality rate is high in premature and immunocompromised infants (*1*,*2*). Multilocus sequence typing (MLST) is a powerful tool for effectively identifying and discriminating different *Cronobacter* strains. Specific sequence types (STs) and clonal complexes are closely related to infections (*3*).

Compared with MLST, CRISPR (clustered regularly interspaced short palindromic repeats) typing is superior for distinguishing similar strains (*4*). *C. sakazakii* ST64, the major ST in food samples, was further divided into 2 sublineages based on CRISPR diversity (*5*). We report a *C. sakazakii* ST64 strain that caused necrotizing enterocolitis in a neonate in China and further examine its origin and phylogenetic relationship with ST64 strains based on CRISPR diversity and whole-genome single-nucleotide polymorphism (wgSNP).

## The Study

This study was approved by the Ethics Committee of Guangzhou Women and Children’s Medical Center (Guangzhou, China; no. 2016081029). Experiments were performed at the Institute of Microbiology, Guangdong Academy of Sciences, and analyses and manuscript preparation were completed at Guangdong University of Technology.

On April 28, 2019, a 17-day-old male neonate born with severe congenital heart disease and perioral cyanosis for 2 hours was hospitalized in a children’s hospital in Guangzhou, China. The patient had necrotizing enterocolitis symptoms develop on May 6 and was given meropenem and metronidazole as antiinfection therapy. However, his symptoms did not improve, and intestinal perforation and peripheral hydrocephalus developed a few days later. Despite the efforts of the doctor, the patient died.

We identified a *Cronobacter* species isolated from ascites by using an automated VITEK 2 Compact system (bioMérieux, https://www.biomerieux.com). An isolate, GZfs, was identified as *C. sakazakii* ST64 of serotype O2. This ST has not previously been reported to cause neonatal necrotizing enterocolitis (*6*). *C. sakazakii* GZfs were susceptible to almost all antimicrobial drugs tested, except cephalothin (Appendix Table). We sequenced genomic and plasmid DNA by using the PacBio RS II (Pacific Biosciences, https://www.pacb.com) and HiSeq (Illumina, https://www.illumina.com) platforms, assembled, and annotated as described (*1*,*7*). *C. sakazakii* GZfs had a single circular chromosome, 4.2 Mb, 57.11% GC content, and 2 plasmids (denoted as pFS1, 115,925 bp, 57.09% GC; and pFS2, 110,391 bp, 50.10% GC) (GenBank accession nos. CP123201‒3).

In our previous study, we divided *C. sakazakii* ST64 strains into 2 CRISPR sublineages, a and b, and compared antimicrobial drug resistance profile strains in sublineage a with strains in sublineage b (*5*). To explore the origin of this pathogenic strain and its phylogenetic relationship with other *C. sakazakii* ST64 strains, we performed whole-genome sequencing of 9 ST64 strains (GenBank accession nos. JARUQD000000000‒ L000000000) and downloaded all ST64 strains with whole-genome sequences from the *Cronobacter* PubMLST database (https://pubmlst.org/organisms/cronobacter-spp) and GenBank genome databases. We provide antimicrobial drug resistance results of 14 food-source ST64 strains (Appendix Table).

After deleting all poor-quality sequences and duplicate strains, we used 66 whole-genome sequences for further analyses. We extracted CRISPR arrays and spacers from those sequences and assigned CRISPR type (CT) numbers to 55 strains with intact CRISPR arrays, according to methods from our previous study (*4*). All ST64 strains had the same 2 spacers: CRISPR3, which was not detected in our previous study because of the lack of *cas* genes (*7*), and CRISPR3, which was not useful for CT in this ST. There were 25 CTs, including 17 new, and we identified *C. sakazakii* GZfs as a new type of CT197 (Appendix). Based on spacer composition, GZfs belonged to CRISPR sublineage b. However, no other strain was found to have an identical spacer profile.

**Figure 1:**
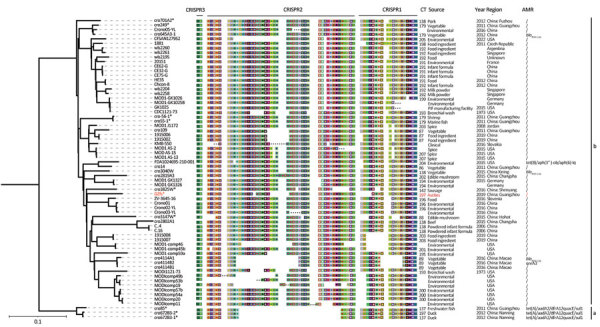
Multilocus sequence typing phylogenetic tree based on whole-genome sequencing single-nucleotide polymorphisms of a *Cronobacter sakazakii* ST64 strain from a fatal case of necrotizing enterocolitis in a 17-day-old male neonate, China, compared with reference strains. Asterisks indicate newly sequenced strains in this study; red text indicates isolate from the neonate. CRISPR spacers arrangement, CT, source, year, region and genes are listed next to corresponding strains. Color schemes in CRISPR arrays are provided at the spacer level to illustrate differences among strains by using CRISPRStudio software (https://www.semanticscholar.org). Ellipsis in spacers indicate partial CRISPR arrays without determined end (incomplete CRISPR arrays). Scale bar indicates nucleotide substitutions per site. AMR, antimicrobial resistance; CR, CRISPR type; CRISPR, clustered regularly interspaced short palindromic repeats; ST, sequence type.

We calculated the wgSNP of ST64 strains by using Harvest software (*8*) and extracted those strains by using SNP-sites software (*9*). We constructed a maximum-likelihood phylogenetic tree by using FastTree software (*10*) and edited in iTOL (*11*). We used a Bayesian phylogenetic approach to estimate the nucleotide substitution rates and divergence times of *C. sakazakii* ST64 according to a previous study (*7*). The maximum-likelihood tree based on the wgSNPs of ST64 strains also showed 2 distinct phylogenetic clusters in accordance with the CRISPR sublineages (Figure 1). The strains in sublineage a were all from food sources.

Sublineage b contained more strains and diverse sources. Moreover, *C. sakazakii* GZfs and all clinical source strains (*C. sakazakii* KMB-550, MOD1-1121-73, and CDC1121-73) in public databases belonged to this cluster. *C. sakazakii* MOD1-1121-73 and CDC1121-73 were isolated from bronchial washes; there was no other patient or disease information regarding those clinical strains.

**Figure 2:**
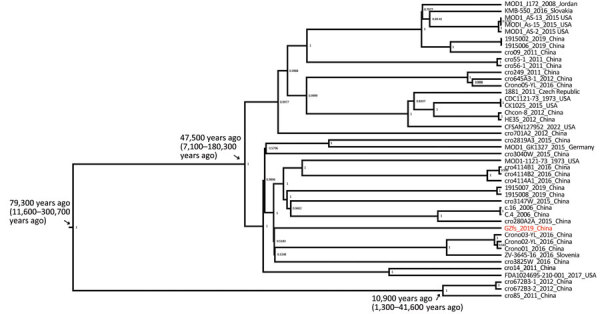
Timed phylogeny in maximum clade credibility tree of *Cronobacter sakazakii* ST64 strain from a fatal case of necrotizing enterocolitis in a 17-day-old male neonate, China, compared with reference strains. Red text indicates isolate from the neonate. Numbers along branches are bootstrap values. Posterior probabilities are shown in the nodes. ST, sequence type.

The genome-wide substitution rate of *C. sakazakii* ST64 was estimated to be 2.3 × 10^6^ substitutions/site/year (95% CI 1.0 × 10^7^–5.3 × 10^6^ substitutions/site/year). According to the maximum clade credibility (MCC) tree (Figure 2), the likely most recent common ancestor of CRISPR sublineage b was 47,500 (95% CI 11,600–300,700) years ago, earlier than for sublineage a, which was 10,900 (95% CI 1,300–11,600) years ago. *C. sakazakii* GZfs had a relatively close phylogenetic relationship with the food-source *C. sakazakii* strain ZV-3645-16 in Slovenia; environmental strains *C. sakazakii* Crono01, Crono02-YL, and Crono03-YL; and the food-source strain *C. sakazakii* cro3825W in China (Figure 1, Figure 2). This finding indicates a close environment food-clinic relationship in dissemination.

We identified acquired drug resistance genes by using ResFinder 2.1. (https://cge.cbs.dtu.dk/services/ResFinder-2.1). All 3 strains in sublineage a acquired the antimicrobial resistance (AMR) genes *tet(A)*/*aadA2*/*dfrA12*/*qacE*/*sul1*, in accordance with their resistance to tetracycline and trimethoprim/sulfamethoxazole (Figure 1; Appendix Table). Five strains in sublineage b had AMR genes; 3 strains (*C. sakazakii* cro645A3-1, cro3040W, cro4114A1) and 1 strain (*C. sakazakii* cro4114B2) isolated from vegetables harbored the AMR genes *bla*_TEM-116_ and *qnrA3*. Four strains were susceptible to all tested antimicrobial drugs (Appendix Table). One environmental strain, *C. sakazakii* FDA1024695-210-001, had *tet(B)*/*aph(3′′)*-*Ib*/*aph(**6**)*-*Id* genes. A total of 92.1% (58/63) strains in sublineage b lacked AMR genes. All 4 clinical strains, including *C. sakazakii* GZfs, did not have AMR genes. In a previous study, *C. sakazakii* ST494 strain was sensitive to all antimicrobial drugs used for treatment; however, the patient died (*12*). Those results suggested that AMR might not be the major reason for the high case-fatality rate associated with this pathogenic infection. Both *C. sakazakii* ST494 and ST64 did not belong to the common pathogenic clonal complex. Enhanced surveillance and pathogenesis research of this organism are warranted.

The virulence genes in *C. sakazakii* remain unclear (*13*), and 2 T6SS and 1 prophage on the chromosome of GZfs might contribute to pathogenicity. The capsular profile of the GZfs was determined to be K1:CA1, as in our previous study (*1*), and plasmid pFS1 was closely related to the IncFIB-type virulence plasmid pESA3 identified in pathogenic *C. sakazakii* strains and pGW2 in *C. sakazakii* GZcsf-1, which causes meningitis in neonates (*1*,*14*). More attention should be given to the study of virulence and pathogenesis.

## Conclusions

We report 1 *C. sakazakii* ST64 strain, GZfs, causing fatal neonatal necrotizing enterocolitis in China that did not belong to the previously identified common pathogenic clonal complexes or STs (*3*). It belongs to the ancient, widespread, and antimicrobial drug‒sensitive CRISPR cluster b of ST64. AMR might not be the major reason for the high case-fatality rate for this pathogen. Public health would benefit from identification of virulence genes and pathogenic mechanisms of *C. sakazakii*.

Appendix Additional information on fatal necrotizing enterocolitis in neonate caused by *Cronobacter sakazakii* sequence type 64 strain of CRISPR sublineage b.
