# Supplementary material for: Fatal Necrotizing Enterocolitis in Neonate Caused by Cronobacter sakazakii Sequence Type 64 Strain of CRISPR Sublineage b
Source: Emerg Infect Dis. 2023 Sep;29(9):1917–20. doi: 10.3201/eid2909.230537 (PMC10461654; doi:10.3201/eid2909.230537)
Supplement: Appendix — Additional information on fatal necrotizing enterocolitis in neonate caused by Cronobacter sakazakii sequence type 64 strain of CRISPR sublineage b. [file 23-0537-Techapp-s1.pdf]

Article https://doi.org/10.3201/eid2909.230537

EID cannot ensure accessibility for supplementary materials supplied by authors. Readers who have difficulty accessing supplementary content should contact the authors for assistance.

Fatal Necrotizing Enterocolitis in Neonate Caused by *Cronobacter sakazakii* Sequence Type 64 Strain of CRISPR Sublineage b

Appendix

Appendix Table. Antimicrobial drug susceptibility profiles

		Antimicrobial susceptibility (KB)[#]								
Antimicrobial group	Antibiotic	GZfs[*]	cro3147W[*]	cro701A2[*]	cro3825W[*]	cro249[*]	cro55-1[*]	cro56-1[*]	cro672B3-1[*]	cro672B3-2[*]
Penicillins	Ampicillin	S	S	S	S	S	S	S	S	S
	Ampicillin/sulbactam	S	S	S	S	S	S	S	S	S
	Amoxicillin/Clavulanic	S	S	S	S	S	S	S	S	S
Cephalosporins	Cefepime	S	S	S	S	S	S	S	S	S
	Ceftriaxone	S	S	S	S	S	S	S	S	S
	Cefazolin	S	S	S	S	S	S	S	S	S
	Cephalothin	R	R	R	S	S	S	S	S	S
Aminoglycosides	Tobramycin	S	S	S	S	S	S	S	S	S
	Gentamicin	S	S	S	S	S	S	S	S	S
	Amikacin	S	S	S	S	S	S	S	S	S
Quinolones	Ciprofloxacin	S	S	S	S	S	S	S	S	S

Antimicrobial group	Antibiotic	Antimicrobial susceptibility (KB)#								
		GZfs*	cro3147W*	cro701A2*	cro3825W*	cro249*	cro55-1*	cro56-1*	cro672B3-1*	cro672B3-2*
Carbapenems	Imipenem	S	S	S	S	S	S	S	S	S
Sulfonamides	Trimethoprim/sulfameth-oxazole	S	S	S	S	S	S	S	R	R
Monobactams	Aztreonam	S	S	S	S	S	S	S	S	S
Amphenicols	Chloramphenicol	S	S	S	S	S	S	S	S	S
Tetracyclines	Tetracycline	S	S	S	S	S	S	S	R	R

Antimicrobial group	Antibiotic	Antimicrobial susceptibility (KB)#							
		cro85*	cro2819A3	cro14	cro109	cro645A3-1	cro2802A1	cro3040W	cro4114A1
Penicillins	Ampicillin	S	S	S	S	S	S	S	S
	Ampicillin/sulbactam	S	S	S	S	S	S	S	S
	Amoxicillin/Clavulanic	S	S	S	S	S	S	S	S
Cephalosporins	Cefepime	S	S	S	S	S	S	S	S
	Ceftriaxone	S	S	S	S	S	S	S	S
	Cefazolin	S	S	S	S	S	S	S	S
	Cephalothin	R	R	S	S	S	R	S	S
Aminoglycosides	Tobramycin	S	S	S	S	S	S	S	S
	Gentamicin	S	S	S	S	S	S	S	S
	Amikacin	S	S	S	S	S	S	S	S
Quinolones	Ciprofloxacin	S	S	S	S	S	S	S	S
Carbapenems	Imipenem	S	S	S	S	S	S	S	S
Sulfonamides	Trimethoprim/sulfameth-oxazole	R	S	S	S	S	S	S	S
Monobactams	Aztreonam	S	S	S	S	S	S	S	S
Amphenicols	Chloramphenicol	S	S	S	S	S	S	S	S
Tetracyclines	Tetracycline	R	S	S	S	S	S	S	S

Antimicrobial group	Antibiotic	Antimicrobial susceptibility (KB)[#]	
		cro4114B1	cro4114B2
Penicillins	Ampicillin	S	S
	Ampicillin/sulbactam	S	S
	Amoxicillin/Clavulanic	S	S
Cephalosporins	Cefepime	S	S
	Ceftriaxone	S	S
	Cefazolin	S	S
	Cephalothin	S	S
Aminoglycosides	Tobramycin	S	S
	Gentamicin	S	S
	Amikacin	S	S
Quinolones	Ciprofloxacin	S	S
Carbapenems	Imipenem	S	S
Sulfonamides	Trimethoprim/sulfameth-oxazole	S	S
Monobactams	Aztreonam	S	S
Amphenicols	Chloramphenicol	S	S
Tetracyclines	Tetracycline	S	S

[#] The results are interpreted using the Enterobacteriaceae data from the Clinical and Laboratory Standards Institute (CLSI).

* These strains are newly sequenced, the genome sequence of other strains have been reported in our previous stud

Dataset Code of CRISPR types (CTs), order of CRISPR array spacers, and dictionary of CRISPR spacers in *C. sakazakii* ST64

CT	CRISPR1-Serial number	CRISPR2-Serial number	CRISPR3-Serial number	CRISPR6-Serial number
82	58	34	2	
83	58	35	2	
~~84*~~	~~58~~	~~33~~	~~2~~	
~~85*~~	~~58~~	~~31~~	~~2~~	
86	59	31	2	
87	60	32	2	
88	61	30	2	
89	62	33	2	
137	58	111	2	
138	58	31	2	
141	97	111	2	
142	98	112	2	
179	57	31	2	
180*	58	139	~~2~~	
191	57	150	2	
192	58	150	2	
193	58	151	2	
194	58	153	2	
195	58	154	2	
196	97	31	2	
197	134	149	2	
198	135	150	2	
199	136	150	2	
200	137	31	2	
201	138	152	2	
202	139	153	2	
203	138	31	2	
204	59	158	2	
205	57	159	2	
206	60	31	2	
207	60	157	2	

Some CT codes were modified for CRISPR3 was omitted in the previous study.

* These CTs (n = 3) after the addition of CRISPR3 serial number were the same as the existing CTs, they were canceled in the new CTs code table.

Order of CRISPR array spacers

CRISPR1-Serial number	CRISPR1-spacers-order (key-order)
57	209,133,132,131,130,129,128,127,126,126,125,124,123,122,121,94,93,514,92
58	209,133,132,131,130,129,128,127,126,125,124,123,122,121,94,93,514,92
59	133,132,131,130,129,128,127,126,125,124,123,122,121,94,93,514,92
60	209,133,132,131,130,129,128,127,126,124,123,122,121,94,93,514,92
61	131,130,129,128,127,126,122,121,94,93,514,92
62	209,133,132,131,130,129,128,121,94,93,514,92
97	209,133,132,131,130,129,128,127,125,124,123,122,121,94,93,514,92
98	209,133,132,131,130,127,126,125,124,123,122,121,94,93,514,92
134	130,129,128,127,126,125,124,123,122,121,94,93,514,92
135	132,131,130,129,128,127,126,126,126,125,124,123,122,121,94,93,514,92
136	209,133,132,131,130,129,128,127,126,126,125,124,123,122,121,93,514,92
137	132,131,130,129,128,127,126,125,124,123,122,121,94,93,514,92
138	209,133,126,125,124,123,122,121,94,93,514,92
139	209,125,124,123,122,121,94,93,514,92

CRISPR2-Serial number	CRISPR2-spacers-order (key-order)
30	177,176,175,174,173,172,171,170,169,168,167,166,165,164,163,162,161,160,159,332,158,157,156,158,157,156,155,154,152,151,150,149,148,147,146,145,144,143,142,49,48,45,28
31	180,179,178,177,176,175,174,173,172,171,170,169,168,167,166,165,164,163,162,161,160,159,332,158,157,156,155,154,153,152,151,150,149,148,147,146,145,144,143,142,49,48,45,28
32	180,179,178,177,176,175,174,173,172,171,170,169,168,167,166,165,164,163,162,157,156,155,154,153,152,151,150,149,148,147,146,145,144,143,142,49,48,45,28
33	180,179,178,177,176,175,174,173,154,153,152,151,150,149,148,147,146,145,144,143,142,49,48,45,28
34	180,179,178,177,176,175,174,173,172,171,179,169,168,167,166,165,164,163,162,161,160,159,332,158,157,156,152,151,150,149,148,147,146,145,144,143,142,49,48,45,28
35	180,179,178,177,176,175,174,173,172,171,170,169,168,167,166,165,164,163,162,161,160,159,332,158,157,156,158,157,153,155,154,153,152,151,150,149,148,147,146,145,144,143,142,49,48,45,28
111	180,179,178,177,176,175,174,173,172,142,49,48,45,28
112	180,179,178,177,176,175,174,173,172,171,170,169,168,167,166,165,164,163,162,161,160,159,332,158,157,156,154,153,152,151,150,149,148,147,146,45,28

CRISPR2-Serial number	CRISPR2-spacers-order (key-order)
149	180,179,178,177,176,175,174,173,172,171,170,169,168,167,165,164,163,162,161,157,156,155,154,153,152,151,150,149,148,147,146,145,144,143,142,49,48,45,28
150	180,179,178,177,176,175,174,173,172,171,170,169,168,167,166,165,164,163,162,161,160,159,332,158,157,156,155,154,153,152,151,150,149,147,146,145,144,143,142,49,48,45,28
151	180,179,178,177,176,175,174,173,172,171,170,169,168,162,161,160,159,157,156,155,154,153,152,150,149,148,147,146,145,144,143,142,49,48,45,28
152	180,178,177,176,175,174,173,172,171,170,169,168,167,166,165,164,163,162,161,160,159,332,158,157,156,155,154,153,152,151,150,149,148,147,146,145,144,143,142,49,48,45,28
153	180,179,178,177,176,175,174,173,172,171,170,169,168,167,166,165,164,163,162,161,160,159,332,158,157,156,155,154,153,152,151,149,148,147,146,145,144,143,142,49,48,45,28
153	180,179,178,177,176,175,174,173,172,171,170,169,168,167,166,165,164,163,162,158,157,156,155,154,153,152,151,150,149,148,147,146,145,144,143,142,49,48,45,28
154	180,179,178,177,176,175,174,173,172,171,170,169,168,167,166,165,164,163,162,158,157,156,155,154,153,152,151,150,149,148,147,146,145,144,143,142,49,48,45,28
157	180,179,178,177,176,175,174,173,172,171,170,169,168,167,166,165,164,163,162,161,160,332,158,157,156,155,154,153,152,151,150,149,148,147,146,145,144,143,142,49,48,45,28
158	180,179,178,177,176,175,174,173,172,171,170,169,168,167,166,165,164,163,162,161,160,332,158,157,156,155,154,153,152,151,150,149,148,147,146,145,144,143,142,49,48,45,28
159	180,179,178,177,176,175,174,172,171,170,169,168,167,166,165,164,163,162,161,160,159,332,158,157,156,155,154,153,152,151,150,149,148,147,146,145,144,143,142,49,48,45,28

CRISPR3-Serial number	CRISPR3-spacers-order (key-order)
2	164,1

Dictionary of CRISPR1 spacers

key	AnnotatedSpacer	sequence
92	sak1–92	CGGTGATGCACCTCGCCGCGATAGATGATCAA
93	sak1–93	CGACGTTTTTACCTACACCGTGCAAATACTGGC
94	sak1–94	ACGGCGACGCTTGTCAGCCCGCCCGAAGGCGT
121	sak1–121	TTGCCGAACGCCACCCAGATAATTTCCGTGCC
122	sak1–122	AAATCTGCATGTTCTGCGGCGAGCCAGCATCG
123	sak1–123	AACCCGGCACTGACAGCCGCCAATGAGTGTAT
124	sak1–124	GTTAATTGCCCGCAGTGCGGGCACCTTTTTGA
125	sak1–125	CCGAGCGCCTTTACATCCTTGCCAGTCGGCTC
126	sak1–126	CGGCGGCCGGGTGCCGTGACTTATTGGGCGTA

key	AnnotatedSpacer	sequence
127	sak1–127	GTACCGCCGCCAGTCTGAGTTGTATTTCTGCA
128	sak1–128	CAACGATTTATCCAGGACGAAGTACACAAAAA
129	sak1–129	GACTCGTTGCTTGTCATTCAGCGGGCATTCAC
130	sak1–130	CAGCATAACCGGATCTGTCGTCTGGCCGTTCA
131	sak1–131	AGAAAGATCAAAATTGCCGGCAACCAATTCTT
132	sak1–132	CGCGATATTATGATCATCACCGGACGGCGCCC
133	sak1–133	AAAGACGGATCCGACATGCGCACGCGAAAACA
209	sak1–209	ATATCATCCCGTCCTGCCTGTCCGTTCACCGT
514	sak1–514	CCGCCTGCGTCCTAACCGGCCTGTAGGCTTTT

Dictionary of CRISPR2 spacers

key	AnnotatedSpacer	sequence
28	sak2–28	ATCGCCAGTATTTTCAACGTGCCGGCGCACAT
45	sak2–45	TTTTCGAAATTGAGCATATTTAACCTATGATT
48	sak2–48	GCGCGACGTATCGCACCGTTGCGCAGGATACC
49	sak2–49	GCCATCGGACGCTTCTGCCAATCTTAACAGGA
142	sak2–142	GGTCATGACGGAAGAGGATGAAGAGGACGCCG
143	sak2–143	GTGTGAGACTTTTGCGCTGCCTAACTTGATCG
144	sak2–144	CCACAGCGCCGCCCTCGCCGGCGGCCGAGTAT
145	sak2–145	TGTACGGGGTAAGGCTGCACCGCATACGTGCG
146	sak2–146	ATTAATATTCATCCTGAACCAGTTAACACCCC
147	sak2–147	ATCGCCTTCGACAGGTCGCCAGGTTCGTCGTA
148	sak2–148	CAGGCGCGCGGTTACGCCTGGGCGGAGAAATA
149	sak2–149	TCGTCGAGGGTTATTTGCATGGCGGCTCGGGG
150	sak2–150	GAGGAACGCCGCCAATTTCAGGAATGTATTTT
151	sak2–151	GGTGTGAAGCTGTTAAGCGTTTTTGAAGATCT
152	sak2–152	TTCGACGATCTCCCCGTCGGCGTGAATGTTCG
153	sak2–153	CATCGGCGTCGCTTGTTCGAGCGCGTCCATCA
154	sak2–154	TCAGTTTCCCGGAGGCGAGCGCCCCTTCAAGC
155	sak2–155	GCTGCGCGACTCGCTGGCCCGTCTCGATACCA
156	sak2–156	ATGTGCCAGAAGTGAAAGCGGCTATCAAACGG
157	sak2–157	GCCGGAACCGGCGCGGAACTGAGGCAGGCATA
158	sak2–158	TGCTGTTATCAGCACTCGTCTGGAACTGGATA
159	sak2–159	CTTACCGCTGGCTGGCAGAGCAGCTCGGCATC
160	sak2–160	TCTGCCGCCCCGGTGCCCAGCGGCCGGACGTG
161	sak2–161	GCAAACTTTCAGTCACTGCAATCGCGTTTATC
162	sak2–162	CATGCGGCCATCGTCGTCAGTGCGCTGTATTC
163	sak2–163	CGCAATCCAAAAATACTGTATATTTGTACAGT

key	AnnotatedSpacer	sequence
164	sak2–164	CCGCTGGTGGGCGTGGGGTGGATATCCGGGCG
165	sak2–165	CGCCTGCGGGCTGTGTCATTACTGGCGTGAAA
166	sak2–166	TCAGGCGGTGTCAGTGGCCGCCTTCACAAGCA
167	sak2–167	CCCGCCGTTCTCGTATTTATTTTTAAATGCCG
168	sak2–168	GCTTCCTGCGTTTTACTGCCTGATAAATCCTG
169	sak2–169	CGTAACGAACTACTGCAATCCAGCGGTTATTG
170	sak2–170	GCCATGAGAACGCCAAAGGCATGTCCGTCACT
171	sak2–171	CGATGTGGGCGGCGGGCGCCACGCGCGTAACG
172	sak2–172	TACACCCGAGACGGGTTTAACAGCAGGTGGGA
173	sak2–173	AGGGTTGAGGCATTCGTCATTGAGGCGCTCCA
174	sak2–174	AATGACCAAAAAAACGCAGACTTTTTTCTTGC
175	sak2–175	AGTTGGTCGCCGAGCGACGCCGCCTGCCGGTC
176	sak2–176	AACTTTCCCGCGATTACAGCCAGGTGAGCTAC
177	sak2–177	CCCCTTTGTTTTACCGAGGCTAAATGCCCAGC
178	sak2–178	AACCGGATAGACAGTTAAATACCAACTATCTA
179	sak2–179	CAACTCGCACACGTCAGCGGAGGAACGCGCTC
180	sak2–180	GAAAAACAGGTGGGTTTTGCCTGTGAGTGTGA
332	sak2–332	GCTCATATTTTTTAAAATTTTTAATATCTAAC

Dictionary of CRISPR3 spacers

key	AnnotatedSpacer	sequence
1	sak3–1	AGACGCGCCGGAAGGGGAGTTGGTGCGCCAGCT
93	sak3–164	CATTGCCTCGATAACCTGCAAATCCCCCTCAC
